# Factors affecting communication in emergency departments: doctors and nurses’ perceptions of communication in a trilingual ED in Hong Kong

**DOI:** 10.1186/s12245-015-0095-y

**Published:** 2015-12-15

**Authors:** Jack K.H. Pun, Christian M. I. M. Matthiessen, Kristen A. Murray, Diana Slade

**Affiliations:** Department of English, The Hong Kong Polytechnic University, Hong Kong SAR, China; The International Research Centre for Communication in Healthcare (IRCCH), The Hong Kong Polytechnic University, Hong Kong; & The University of Technology Sydney, Sydney, Australia; Department of Education, St Antony’s College, University of Oxford, Oxford, UK; Faculty of Arts and Social Science, The University of Technology Sydney, Sydney, Australia

**Keywords:** Interviews, Emergency medicine, Clinician-patient communication, Clinician-clinician communication, Quality of care

## Abstract

**Background:**

This study investigates clinicians’ views of clinician-patient and clinician-clinician communication, including key factors that prevent clinicians from achieving successful communication in a large, high-pressured trilingual Emergency Department (ED) in Hong Kong.

**Methods:**

Researchers interviewed 28 doctors and nurses in the ED. The research employed a qualitative ethnographic approach. The interviews were audio-recorded, transcribed, translated into English and coded using the Nvivo software. The researchers examined issues in both clinician-patient and clinician-clinician communication. Through thematic analyses, they identified the factors that impede communication most significantly, as well as the relationship between these factors. This research highlights the significant communication issues and patterns in Hong Kong EDs.

**Results:**

The clinician interviews revealed that communication in EDs is complex, nuanced and fragile. The data revealed three types of communication issues: (1) the experiential parameter (i.e. processes and procedures), (2) the interpersonal parameter (i.e. clinicians’ engagements with patients and other clinicians) and (3) contextual factors (i.e. time pressures, etc.). Within each of these areas, the specific problems were the following: compromises in knowledge transfer at key points of transition (e.g. triage, handover), inconsistencies in medical record keeping, serious pressures on clinicians (e.g. poor clinician-patient ratio and long working hours for clinicians) and a lack of focus on interpersonal skills.

**Conclusions:**

These communication problems (experiential, interpersonal and contextual) are intertwined, creating a complex yet weak communication structure that compromises patient safety, as well as patient and clinician satisfaction. The researchers argue that hospitals should develop and implement best-practice policies and educational programmes for clinicians that focus on the following: (1) understanding the primary causes of communication problems in EDs, (2) accepting the tenets and practices of patient-centred care, (3) establishing clear and consistent knowledge transfer procedures and (4) lowering the patient-to-clinician ratio in order to create the conditions that foster successful communication. The research provides a model for future research on the relationship between communication and the quality and safety of the patient safety.

## Background

Governments, healthcare organisations and researchers are increasingly recognising the critical role that communication plays in patient safety and in the provision of quality health care, particularly in high stress contexts such as emergency departments. Communication in an Emergency Department (ED) is a key contributing factor to patient safety and satisfaction, as well as clinician satisfaction and retention. The communicative challenges and risks in emergency departments arise directly from the significant and increasing contextual complexity of the emergency department environment. This complexity stems from a number of factors, including 24-h care, the increased demand, the short-term and episodic nature of emergency care and the impact it has on clinician-patient relationships, cross-level and cross-disciplinary teams working together and the linguistic and cultural diversity of both clinicians and patients. In these high stress contexts, there is even a greater burden placed on effective communication to ensure the quality and safety of patient care [1]. Conversely, ineffective communication is a major cause of critical medical incidents in EDs and causes in a significant number of patient grievances [2, 3]. Recent research demonstrates that communicating care is as important as delivering care to patients [4, 11]. In addition, optimal healthcare communication is linked to higher levels of clinician job satisfaction and lower levels of clinician turnover. The research presented in this paper responds to increasing awareness of the importance of patient-centred communication in EDs. This work not only highlights the nature and impact of communication problems but also proposes programme and policy solutions.

Research demonstrates that effective communication in EDs is a crucial factor in determining the quality of care patients receives and the patients’ responses to that care [3, 5–9]. A study on surgical staff within the US National Institutes of Health points out that two significant factors causing critical incidents were “blurred boundaries of responsibility” and “distorted or inhibited communication” [10]. Slade and her colleagues, who examined clinician-patient communication in five EDs across Australia, report that the quality of patient’s care and experience are affected by the contextual complexity of EDs and by the foregrounding of the medical aspects of communication over the interpersonal aspects [4, 11]. A key finding of their research was that communication across the five EDs was rarely patient-centred with very little rapport and empathy being developed between the patients and clinicians. While acknowledging the challenges that affect the development of rapport and empathy, such as the severe constraints on the time that clinicians have face-to-face with patients and the lack of pre-established relationships between patients and clinicians, they argue that this dimension of communication is critical for safe and effective care. The evidence they have of over 80 patient recordings from triage to disposition is that positive interpersonal relationships between clinicians and patients result in a higher degree of patient involvement, which in turn produces better clinical outcomes, such as mutually agreed treatment plans and better patient adherence.

In Hong Kong, research has identified similar concerns with communication issues and problems in EDs. The School of Public Health and Primary Care of the Chinese University of Hong Kong, with support from the Hong Kong Hospital Authority, conducted a patient survey in 25 selected public hospitals (of the more than 5000 patients surveyed, over 65 % were ED patients). This survey showed that communication is in need of significant improvement in public hospitals in Hong Kong, especially in EDs. Specifically, patients expressed their dissatisfaction with the explanations they were given in the ED and their own level of involvement in decision-making about their care and treatment [12].

Similarly, Tam and Lau [13] looked at different types of patient complaints and the levels of patient satisfaction in a Hong Kong ED. In the 71 complaints from 1995 to 1998, nearly half (49 %) of the patients’ dissatisfied comments were about insufficient communication. Patients complained about the poor attitude of the ED staff, the clinicians’ poor communication skills, their impatience during examination and their miscommunication at clinical handover. Tam and Lau suggested that complaints from patients could be reduced if ED clinicians were more aware of patients’ emotional needs. Listening and talking to the patient clearly and thoughtfully were among the key factors that reduced patient complaints.

This research stems from a mixed methods research project in a large, busy, trilingual ED in Hong Kong. The larger project involved a total of 80 h of direct observations, audio-recordings of 10 patient journeys from triage to disposition, a survey of clinicians and 28 in-depth interviews with ED clinicians and hospital management. Thus the project as a whole has been designed to provide a holistic view of patient journeys, based on the different data sets just mentioned (recordings, observations, interviews) and the different forms of analysis, including both ethnographic and linguistic approaches, adopted to illuminate different types of data. The general methodology is thus one of the mixed methods—adopted to enable triangulation. In this paper, the focus is on the interviews; findings from other forms of data are published elsewhere [14, 15]

By analysing the qualitative data from the 28 clinician interviews, the researchers identified the major motifs, or communication factors, that either enhance or impede clinician-patient and clinician-clinician engagements in the ED. Overall, this research highlights the complexity of the flow of communication in EDs, explains the specific factors that contribute to unsatisfactory communication and offers potential solutions to improve patient safety and satisfaction and enhance clinician satisfaction.

## Methods

### Design

This study was conducted according to current ethical guidelines; it was approved by the two ethics committees of the Hong Kong Polytechnic University and the participating hospital. All participating clinicians signed a consent form to be interviewed and audio-recorded. Twenty-eight (28) clinicians working in the ED were interviewed. The interviews were audio-recorded, transcribed, translated and de-identified. The data were then coded with the Nvivo 9 software package, using thematic analysis codes. Table 1 displays the demographic data for these clinicians.

**Table 1:** Demographic background of the doctors and nurses working in the ED

	Nurses	Doctors	Total
Age			
Under 35-year-old	12	4	16
Over 35-year-old	8	4	12
Number of years in ED:			
1–4 years	7	4	11
5–9 years	3	0	3
10 years or above	10	4	14

### Data

This research involved interviews with eight doctors and 20 nurses in this ED. These clinicians were of diverse specialties and levels of seniority. Their work experience ranged from 1 to 25 years. All interviews were conducted in semi-structured mode, which provided each clinician with the time and flexibility to express his/her opinion and share experiences related to communication in this ED. Table 2 displays the professional background of the clinicians.

**Table 2:** Background of the interviewed doctors and nurses in the ED

Nurses		Doctors	
(Male 11, female 9)	Total: 20	(Male 8, female: 0)	Total: 8
Register nurse	13	Doctor specialists	2
Nurse specialists	2	Residents	4
Ward managers	3	Management	2
Nurse officers	2		

### Data analysis

For this research, data analysis involved the careful examination of all transcribed data from the interviews. We identified recurring communication issues, explored the connections between issues, evaluated the seriousness of the problems and weighed the overall impact of these factors on clinician-patient and clinician-clinician communication. We engaged in several rounds of comparing, sorting, recoding and looking for issues and connections in the data. From this process, we identified three main motifs, which were different types of communication problems in the ED. The first motif was experiential in nature (i.e. communication factors related to medical processes and procedures in EDs). The second was interpersonal (i.e. clinician-patient and clinician-clinician communication). The third was the contextual factors in ED communication (i.e. clinician-patient ratio, patient expectations, etc.). By delineating these three main types of communication issues, and by pinpointing two key problems within each type, the researchers revealed how communication in EDs is simultaneously affected by experiential, interpersonal and contextual factors. Within each of the three types of communication issues, there are two specific concerns:

1. The experiential parameter (i.e. medical processes and procedures)
  Inadequate transfer of medical knowledge and information (e.g. triage, handover);
  Discrepancy between information given to doctors and to nurses.
2. The interpersonal parameter (i.e. clinicians’ engagements with patients and other clinicians)
  Lack of focus on developing empathy and rapport with the patient;
  Barriers across the clinicians’ disciplines and levels of seniority.
3. Contextual factors (patient and staffing numbers, patient expectations)
  Time pressures (i.e. high number of patients, staff shortages and long working hours);
  High patient expectations.

In short, communication in EDs is an intricate construction that contains a number of structural weaknesses; these areas of fallibility place patient safety and satisfaction at risk and they increase clinician burnout.

In addition to the communication problems outlined above, we identified a major issue with the clinicians’ constant translation between spoken Cantonese, or Mandarin (used in discussion with patients in the ED), and written English (used in medical notes). This process of translation occurs continually in a trilingual ED, as doctors and nurses move through patient triage, handover and treatment. Clinicians use Cantonese and English, and occasionally Mandarin, to shift between conversational vocabulary, technical terms, names of medications and treatment plans. This process generates a high possibility of misinterpreting, omitting or altering crucial medical information, thereby placing patients at risk. Due to the magnitude and complexity of this translation issue, we have not focused on this problem within this paper; instead, the issue of translation will be addressed in another paper by the same research team.

The findings from this research project not only identified three main types of communication issues (the experiential parameter, the interpersonal parameter and the contextual factors) but also demonstrated the connections between these issues. The following section provides precise data from the interviews to illustrate each of the above communication problems. This discussion also reveals links between these interwoven communication challenges.

## Results

In the high-stressed, time limited context of the ED, communication is complex, nuanced and interrupted, fragmented, rushed and consequently error prone. Patients experience extensive waiting times and often have as little as 6 min with the doctors. As a result, patients often feel that the Doctor(s) had not adequately listened to them and consequently did not agree with or understand their diagnoses or their treatment plan. More than 50 % of the patients we recorded left feeling anxious and confused about the nature and impact of their condition. Yet clinicians argued that it was difficult to communicate with patients at length, to explain their diagnoses, treatments and prognoses frantic, fragmented and unpredictable nature of EDs. Doctors and nurses feel enormous pressure to perform their clinical tasks efficiently and therefore they argue with very little, if any, time to attend to the interpersonal needs of the patients. The clinicians interviewed for this research admitted that it was difficult to attend to the interpersonal aspects of communication, due to the contextual constraints and that they simply focused on treating the patient’s specific medical condition [12, 13]. The combination of these expectations and limitations creates a highly complex, challenging environment for communication.

A key finding of our analyses of the 10 patient journeys [14] as well as Slade’s research in five EDs in Australia [4] is that establishing a positive interpersonal relationship with the patient is not just so the patient “feels good” about his or her experience in the emergency department. The evidence we have from the many recorded interactions is that the development of rapport and empathy between clinicians and patients result in a higher degree of patient involvement, which in turn produce better clinical outcomes, such as mutually agreed treatment plans and better patient compliance.

### The experiential parameter

#### Inadequate transfer of medical information

This type of communication problem involves processes and procedures in an ED. Due to the interdisciplinary nature of healthcare in EDs and the number of different clinicians patients may see, there is the rapid exchange of information about patients’ diagnoses, conditions and treatment. Because patients often present at an ED without any medical records, clinicians rely heavily on the medial records created within the ED. The interview data for this project identified two key issues regarding the transfer of medical information: (a) omissions and inconsistencies in medical records and (b) inadequacies in triage and handover practices. Each of these medical aspects of communication was seen as impacting patient safety and satisfaction in the ED.

A comprehensive understanding of patients’ conditions and medical history is crucial to practising quality healthcare, especially in an urgent care context. Yet clinicians admitted that often a patient’s notes are consisted of short, dense and illegible words and/or characters that could result in confusion, omission or alteration of medical information. In addition, clinicians may lack the information they need to adequately perform triage and handover.

For some cases, the [medical] info wasn’t entered into the system; for example, family doctors or, say, visiting medical officers, or say doctors from nursing homes. We don’t have such records [in the system]. But their information is quite valuable to us. [They] know what the patient is usually like…

While some of the clinicians stated that they did not think handovers were problematic in the ED, many other clinicians gave clear descriptions of inadequate, inconsistent communication engagements at handover. The data revealed that there are no standardised practices for conducting clinical handovers in this ED; handovers may be spoken or written, informal or formal, and they may be omitted altogether.

Our colleagues basically understand [the procedures]… There’s no need to [handover], there’s no need cause there’s so many things [to deal with]. Say a doctor dealt with five cases, we have ten doctors a day, that’s 50 cases, it’s just impossible [to do handovers].

In addition, clinicians reported that they often handle patient care via pattern recognition, rather than by receiving clear, thorough information via handover or by asking questions of the patient or other clinicians.

This is not really about what you read [in the medical notes]; this is a pattern recognition. [When you] read the notes, you’re not reading each and every word. Rather, you expect what s/he’s going to write. I mean, then we use the pattern to match things up. “Eh? This looks like, say, abrasion on the right hand and such” or this is a laceration on the left hand. I mean, this is the way things go. Such is the departure point to read notes, actually it’s easier to read.

In this sense, detailed medical records were seen as complementary and optional, not essential and compulsory. However, neither the process of over-focusing on medical details nor the act of over-generalising about overarching symptoms generates sufficient understanding of a medical condition or treatment. A few clinicians admitted that this inconsistent, casual approach to handovers sometimes resulted in mistakes.

Hmm, sometimes, e.g. last week our patient needed to go up to operation theatre directly from our trauma room. Our staff – the medical officer prescribed the pre-medication to the patient, but this pre-medication would be given to the patient inside the operation theatre. Our staff forgot to handover the drugs to the operation theatre, the staff in the operation theatre. And the drugs came back to our department without handover to the other department. Ah, it will waste time. Although the patient will receive such medication, but just a little bit time delay for that.

Because both the triage stage as well as the clinical handovers within the ED and at discharge represent crucial moments in the provision of healthcare services, these two points of transition deserve further research attention, as discussed below.

#### Discrepancy between information given to doctors and nurses

In relation to explaining and updating information for patients, some nurses were concerned that they may not possess the information they need to answer patients’ enquiries.

[The patient] just picks out anyone in random and ask questions. Then you go, “Mm… actually I’m not clear on this. Gotta ask. Tell me your name and let me check your records first.” But after checking the records, seems like the doctor didn’t mention that. Perhaps the doctor has told the patient, “In a moment we may put you on a saline drip. But [according to] the medical notes, x-ray isn’t done and this treatment wasn’t jotted down yet and so on. Perhaps. So actually the doctor hasn’t put it down in the medical notes.

As the following quote shows, doctors and nurses sometimes presume that the other party has explained a medical condition, test or treatment to the patient so that patients do not actually receive information on post-consultation treatments and the subsequent steps involved in their patient journeys.

But sometimes, perhaps the doctor thinks us nurses will deal with that [explanation of treatments, but] we think the doctor did. In other words, this information goes missing. Then after the consultation, the patients don’t know what they’re going to do.

Patients approach nurses, at random, with the expectation that the patients’ enquiries may be answered. However, nurses may not have the complete picture, due to lack of access to all the medical notes. Thus, the compartmentalisation of responsibilities between doctors and nurses leads to problems in the transfer of information. In the following quote, a clinician describes the ED as a factory, both physically and semiotically.

In ED, the workflow is little bit like factory, manufacturing line. Just like a factory. Ah, so they [medical personnel] know the job in bit by bit; they know the process in bit by bit. Except some special case like the trauma case [or] the resuscitation case. They will handover in detail. In other cases, they just do what they are doing in the common day.

In addition to discrepancies in access to medical information between different clinicians, there were also variations in how different clinicians described and measured medications. This posed a serious risk in administering treatments.

In some situations, the medical staff, unlike us from ED, they come from, say, ICU [Intensive Care Unit], or other specialties to consult. Their preparation [procedures] and weight [in measuring dosages] are different. So after they give a command, we have to think about how to administer the dosage.

### The interpersonal parameter

The interpersonal aspects of communication involve clinicians’ engagement with both patients and other clinicians in an ED. This subsection of the paper presents clinicians’ varying perspectives on the relative importance of establishing rapport and empathy, combined with the clinicians’ perceived inabilities to engage with these dimensions of healthcare.

As noted above, quality medical care depends on clinicians establishing an effective and respectful interpersonal relationship with patients. The central goal of these interpersonal connections is to improve the quality and safety of the patient experience [4, 16, 17].

#### Lack of focus on empathy and rapport

Brock and Salinsky [18] define *empathy* as “the skills used to decipher and respond to the thoughts and feelings passing from the patient to the physician”. Leach [19] suggests that *rapport* between doctor and patient is a “therapeutic alliance” developed by trust and cooperation with a mutual understanding of patient’s perspective. Empirical research shows that rapport can be positively reflected by a high level of synchrony in monitoring doctor and patient’s heart rates during psychotherapy consultations [20]. Empathy has been shown to increase self-efficacy and emotional distress during oncological consultations [21]. Empathy and rapport not only account for doctor’s efficiency in communicating with patients but also enhance patient satisfaction and patients’ overall health outcomes [22, 23]. Slade et al. [4] found substantial evidence demonstrating that the effectiveness of clinician-patient communication is highly dependent on how the clinicians builds and maintains an interpersonal connection with the patient. In general, clinicians’ efforts to develop rapport and empathy are not external, unnecessary tasks; the interpersonal factors are integral to successful healthcare communication.

Doctors and nurses generally think it is important to develop empathy and rapport with patients, but they emphasise that their main priority is patient’s safety, as though these two aspects of medical practice were mutually exclusive. One junior doctor explained this perspective as follows.

Compared to the wards, [empathy and rapport] doesn’t matter as much in ED. Since basically we don’t get to see the patients again after treatment, there’s no rapport, really. We’re just with each other for a few minutes. Even for assessments, it’s just going to take half a day. So relatively speaking–not that it’s pointless [building empathy], but relatively speaking–if the patients trust you, they’ll trust you [more] next time; but it isn’t long-time [patient] care, [empathy] isn’t… compared with other specialties, it isn’t very important.

Despite this viewpoint, current patient-centred practice demonstrates that communicating medical knowledge and building interpersonal relationships should not be interpreted as separate phenomena. Following Slade et al. [11], we argue that both these processes must occur simultaneously and that to “deliver care effectively, clinicians must communicate care effectively” [14].

In addition to the compartmentalising of empathy and rapport, as quoted above, a few clinicians expressed a slightly negative viewpoint about interpersonal skills. These clinicians said that empathy and rapport were time-consuming and less important than technical considerations. These aspects of communication were seen as the privilege of medical staff working in the wards, not in the ED. The following quotes illuminate this viewpoint further.

My opinion is that we are delivering a service to our patients, so they are actually our clients.

Building rapport, a good relationship with patients, is very important. But it is very difficult for our emergency physicians to do this. Okay. Because the time is very limited; and the patient, most of the patient[s] is [are] first seen by you. Okay? The relationship is quite difficult to develop in a very short time, because our workload is very high and the patient [load] is unlimited and also …there is a long queue in ED. I think it’s very difficult

Senior level clinicians expressed the issue of developing interpersonal communication skills in the following ways.

Usually we don’t have much time to build up rapport with patients, right? Just a few minutes, how can you build up rapport? [Chuckles] Yeah, you can at most speak for one sentence. Yeah, yeah, how can you build up rapport in that time, right?

I mean you’ll definitely administer treatment first, instead of thinking about this thing [empathy].

I think the time limitations certainly play a part in that ah issue. Particularly in ED…And, so ah, we tend – even in medical field – we tend to stereotype the ED doctor, into very efficient, ah, fast-moving guys. They lack empathy. That is the stereotype.

#### Barriers across different clinicians’ disciplines and levels of seniority

Due to the organisational culture of EDs in Hong Kong, hospital hierarchy was cited as a significant problem. Many junior doctors and nurses were anxious about asking for clarification or confirmation from senior clinicians. Compounding the problem, some of the senior clinicians may not be approachable, thereby deterring the process of questioning and understanding that leads clinicians to make better decisions. Some clinicians elaborated on this point, saying that junior clinicians do not seek advice from senior clinicians because the juniors do not want to appear weak or lose face. One clinician expressed the problem in the following manner.

It’s… up to the staff individually, because some of [the doctors] could be over-confident. Mm, so that’s why we always remind the juniors to consult the seniors whenever you have doubts. But sometimes we also observe that some doctors are over-confident that everything is fine; and e.g. if they look at x-ray, um, they may miss a fracture because they are over-confident. They don’t ask. Um, I think this is still happening.

This barrier to the accurate transfer of medical information steams from an overt focus on hierarchy among medical staff.

Sometimes for the junior nurse, because our–we are their senior, when we ask question “Do you understand that?” They always say “Understand.” But actually they don’t understand. But they don’t want to ask. And they don’t want–they don’t want to lose face… Usually junior don’t want to ask too much questions, especially for the nurse to ask the doctor, they think that they are so foolish. They don’t want to ask too many questions. But ah for nurse to nurse, this scenario… will be much better. But… always like that, if you are too senior, they won’t ask you.

Along these lines, one doctor said that, while it seemed there was sufficient time to establish empathy with patients, there simply was no point in doing so because the doctor would not see these patients again. This perspective shows a lack of awareness of the tenets of patient-centred care, as well as the research that points to the positive relationship between patient-centred practices and better patient outcomes [24–26].

Although the clinicians interviewed were generally aware of the importance of building empathy and rapport with patients, they did not have a shared understanding of what this may entail. This caused clinicians to enact quite different, even random, approaches to establishing empathy and rapport with patients. As previously mentioned, building empathy and rapport is actually integral to ensuring patients’ safety and satisfaction. These communicative process works in tandem.

### Contextual factors

#### Time pressures

Among the most significant contextual variables mentioned by clinicians was time. It was referred to constantly, as a factor in almost all aspects of providing medical services. One key problem was the clinician-patient ratio, which created constant time pressures and made both doctors and nurses feel they should work far more hours than stipulated in their contracts.

Ah, formal hours [are] 44 hours a week. But actually I work, maybe, more than 12 hours every day, including–even this Monday, I–I–I got–I had annual leave, but I still need to work. Heh.

The clinicians also mentioned that, as a result of their long working hours, sometimes they have little to no sleep at night. Consequently, they feel less capable of engagement with patients, feeling less concerned with patients’ needs and just doing the basics.

As a nurse manager, I have to reflect the real situation: [we have] the highest [patient] attendance rate in Hong Kong, but with the lowest nurse manpower.

One key consequence of the staff shortages is that doctors and nurses spend much less time with each patient than they would like to spend (e.g. one triage nurse said she is required to see one patient per minute, or 60 patients per hour). This point highlights the interaction between time pressures and the inadequate transfer of medical knowledge, discussed above. With these time restrictions, clinicians cannot exchange information effectively; they can only provide the most basic information.

But ah because of the time limitation and ah–yes mainly the time; maybe, you will be [referring to] the high attendance, too many patients, and the–the patients feeling that “you don’t want to listen to me”.

These time pressures also affect the clinicians’ abilities to participate in continuing professional education, to relate well to patients and other clinicians and even to look after their own health. Clinicians frequently cite time pressures as the main reason for limited interpersonal communication.

Saving lives is our top priority. Right. These [interpersonal communication skills], to be frank, are time-consuming. I have to sit beside you [the patient] for a long time, “Mm … you must be feeling bad.” It may take a quarter or nearly half an hour for him/her to feel just better… But think about it, a quarter or half an hour, it can be spent on dealing with an emergency case.

With a high attendance rate of 600 patients per day in the ED, each doctor can only spend approximately 5 min on each patient. It is very difficult for them to devote extra time to developing empathy and rapport with patients. Thus, ED doctors and nurses were often stereotyped as fast but brusque decision-makers, who only focus on patient’s physical discomfort, paying minimal attention to their mental needs.

In addition to time pressures, there were other contextual factors (i.e. physical, social and organisational cultural variables) that clinicians believed may decrease the quality of communication in EDs. The physical environment was seen as noisy, overcrowded and uncomfortable for some patients. There was also poor clarity in the public announcement system, which could create confusion.

#### High patient expectations

Many nurses stated that Hong Kong patients often had unrealistic expectations of ED care and this created a “culture of complaint”.

The stress level is a little bit high in every nurse in the department. And, ah, I think, um, nowadays the Hong Kong people, the public request more than before. They expect more than before …

[There is a] higher expectation of the public, of the patient, you know, Hong Kong is a culture of complaint. All patients raise their personal concern.

Some of the patients who come to see the ED, their underlying want, or their expectation, is they want admission….Yes, but their expectation is very high. Or maybe very demanding. We have to see the underlying concern or the idea of them and try to tackle the underlying idea [of] why they want to be admitted to the hospital.

Due to unmet expectations, patients may become agitated, which can manifest itself in problematic behaviours within EDs in Hong Kong.

The time pressures noted above, as well as the experiential and interpersonal factors discussed previously in this section, create a considerable gap between patients’ expectations and the realities of healthcare services in an ED. Overall, it is vital to consider the complicated, overlapping relationship between these three areas of communication in EDs.

## Discussion

### Interweaving interpersonal and experiential factors

As noted in the previous sections, each patient who enters a large, busy, trilingual ED in Hong Kong encounters complex communicative networks involving multiple clinicians and repeated exchanges of medical information. This paper maps out how particular experiential, interpersonal and contextual factors hinder clinicians’ communicative practices and thereby impose risks on patients’ safety, reduce patient satisfaction and detrimentally affect clinician satisfaction. The risks for these communication fractures and errors are high: research shows that communication problems cause avoidable readmissions to hospitals, adverse events (e.g. under- or overmedication and damaging drug interactions) and missed diagnoses, which may have serious consequences, including death [4, 11, 27].

**Fig. 1:**
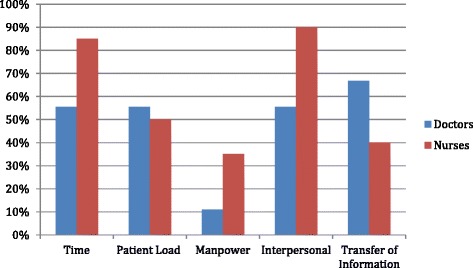
Most frequently cited communication problems in EDs, according to clinicians (*n* = 28)

The clinicians interviewed in this study articulate how the compartmentalisation of roles and processes, and the reluctance to see the interconnected nature of all these factors, impact upon the quality of communication in EDs. Figure 1 demonstrates that, of the top 5 most frequently cited communication problems, only one was an interpersonal factor; the other four problems were experiential or contextual factors. This finding demonstrates how clinicians tend to foreground the experiential factors, while often dismissing the interpersonal aspects of communication. When clinicians recognised the importance of interpersonal factors in communication, they frequently state that these areas were impossible to adequately address in a large, busy, trilingual ED due to contextual factors (e.g. time constraints).

Figure 1 summarises the percentage of doctors and nurses, out of the 28 interviewed for this project, who mentioned the communication problems identified and discussed here. The problems that clinicians highlighted were from all three main types of communication issues:

1. The experiential parameter
  Problems with the transfer of information (doctors 67 %; nurses 40 %)
2. The interpersonal parameter
  Lack of focus on building relationships with patients (doctors 56 %; nurses 90 %)
3. Contextual factors
  Time limitations (doctors 56 %; nurses 85 %)
  High patient loads (doctors 56 %; nurses 50 %)
  Staff shortages (doctors 11 %; nurses 35 %)

Doctors were the most concerned about the transfer of medical information; in mentioning these issues, they emphasised the potential impact on patient safety. Similarly, nurses expressed concern about staff shortages, noting the detrimental effect on the quality of patient care. (In Hong Kong, nursing staff numbers were reduced in public hospitals in recent years [28].) Nurses also voiced their concerns that there was an insufficient focus on developing empathy and rapport with patients.

This paper builds on the premise that a satisfactory patient journey through the ED relies on an effective integration of the experiential, interpersonal and contextual aspects of communication. All of these factors are intertwined; together, they currently deter, or even prevent, clinicians from engaging in effective communication.

Although interdisciplinary and cross-disciplinary communication between clinicians forms the basis of the transfer of medical information during each patient’s journey in the ED, there is a level of ambiguity in clinician’s roles, thereby creating potential confusion and misinterpretation by both clinicians and patients. For example, nurses may be expected to “cover” for doctors without having adequate medical information available. Doctors may assume that patients have been informed of particular issues, but nurses have not had the opportunity to impart this important information. This uneven distribution of patient-related information leaves nurses unable to answer the patients’ queries and doctors are uncertain of the patient’s level of understanding; this, in turn, affects patient safety and satisfaction.

As discussed above, both triage and handover play a critical role in facilitating effective healthcare communication. Yet further research is needed in both these areas, as these points of transition have only recently begun to receive intensive research attention. Handovers, in particular, represent the clinician-patient engagement in which the experiential, interpersonal and contextual aspects of healthcare communication interact to the greatest degree. As a result, handovers are an ideal focal point for future research and programme intervention. By teaching clinicians to conduct consistent, thorough, patient-centred handovers, hospitals may see exponential improvements in all three of these aspects of communication. In the following section, the researchers reiterate key findings and set forth potential solutions to the communication challenges discussed above.

## Conclusions

This study analyses the key healthcare communication issues raised by doctors and nurses working in a trilingual ED. Overall, these communication problems fit into three main categories: the experiential parameter (i.e. procedures and policies), the interpersonal parameter (i.e. clinicians’ engagements with patients and other clinicians) and contextual factors (time pressures and patient expectations). Within each of these three types of communication issues, there were two additional, specific problems that hindered clinician-patient and clinician-clinician communication.

Within the experiential parameter, clinicians reported that the medical information transferred at key points (e.g. triage and handover) could be incomplete or unclear, thereby placing patients at risk. This was due mainly to inconsistent record keeping and inadequate handover procedures. Clinicians also cited problems with organisational culture factors, such as friction between disciplines and different levels of seniority. Clinicians noted that doctors and nurses have different access to medical records; this uneven distribution of patient information creates confusion for both clinicians and patients.

Within the interpersonal parameter, clinicians said that developing empathy and rapport is difficult, if not impossible, in an ED context. Some clinicians admitted they have received minimal training in communication, as well as little exposure to the tenets of patient-centred care and other policy imperatives in the healthcare services. However, research demonstrates that optimal communication should not only focus on the application and transfer of medical knowledge but also on the patients’ interpersonal needs (i.e. desire for empathy and understanding of their condition).

In terms of contextual factors, time pressures were seen as paramount. The high number of presenting patients in this large, busy, trilingual ED, in relation to the insufficient number of clinicians, left doctors and nurses with a very short time for triage and almost no time for handovers. Doctors reported working considerably extended hours, in addition to their contracted work period, resulting in fatigue and stress. Clinicians emphasised that time pressures and long work hours placed a considerable strain on communication.

Following the identification of key issues, as above, this paper offers possible solutions for each area of difficulty in ED communication. These translational research implications focus on practical solutions for clinicians and hospitals to develop and implement. This paper contends that, by improving communication in EDs, hospitals and patients will reap significant benefits.

### Proposed solutions

In the research interviews, clinicians put forward possible solutions for the communication problems in their working environment. Despite acknowledged time and budget constraints, clinicians proposed several suggestions for better communication in EDs.

One potential solution, regarding the experiential factors in communication, involved the standardisation of medical record keeping, via an electronic system. With the help of such a system, information would be quickly and consistently available to all doctors and nurses in the ED, as well as in specialist centres.

In regards to the transfer of information between different disciplines and levels of seniority, a positive development would be a clear requirement to document instructions related to medications, tests and treatments in a written form. This is important because different clinicians may use a range of terms, abbreviations and different ways of measuring and preparing medications. It is therefore crucial that this information is documented in written form and not just stated verbally. One nurse explained her current approach, which is not yet a standard procedure.

I will ask [the staff who issued the command] to write it down, literally, if we just don’t understand. Say the ICU [staff] administers M and M, morphine and midazolam, which are seldom used in ED. We’ll ask him/her to write down the preparation and so on. Then we follow.

In addition, another suggestion was that all communication between disciplines should be accompanied by a statement-confirmation requirement, in spoken or written form. This would be followed by a request for an additional opinion from other personnel if needed. A statement-confirmation requirement would help ensure that all medical information is understood by all clinicians and is therefore transferred accurately.

For the interpersonal parameter, clinicians identified strategies being used currently to enhance patient satisfaction. Most of these strategies involve clinicians shifting to other communication modalities (i.e. body language). Firstly, clinicians could present themselves clearly to patients, which takes only 30 to 60 s and which makes a significant difference to the quality of the interaction with the patient. Second, the clinicians could ask what the patient believes to be wrong, instead of immediately making decisions. Third, the clinicians could listen to patients and treat them with respect, using good manners and a kind, calm voice. One nurse described the benefit of giving patients comprehensive explanations about their situation, especially when patients are agitated about their experience in the ED.

I can’t say [the explanation] takes away his/her anger 100 %, or that s/he doesn’t lose it and stuff; they still have to wait for a long time. But s/he… can lower the extent [of dissatisfaction]. I mean, there are some reasonable folks, you talk to them, they’ll feel better. Things like that.”

Adequate explanations are considered crucial in establishing empathy and rapport, one reason being that the more informed patients are, the more emotionally stable they may be. A number of clinicians explained this point.

If the patients trust you, they’ll trust you [more] next time.

I have my own ways: first of all, I speak slower. And even if I speak fast, I slow down at crucial points. I discovered that patients found it easier to absorb things. When they find things easier to absorb, when they understand what you’re saying, things are better.

In addition, clinicians can make eye contact with patients and present an open, caring facial expression. Without expending significant amounts of time, clinicians can be supportive and ask prompting questions to pinpoint symptoms and possible causes of illness.

Lots of times when the elderly come, they say they’re feeling queasy all over, so you ask, “Where specifically?” Ask if s/he can point out somewhere specific, you can take a look and see if there’re any relevant symptoms… That way, you hope you can get to know the symptoms.

In terms of contextual factors, there are two main changes that could make a significant impact on the quality of communication in EDs. First, hospitals could avoid staffing shortages by ensuring that more doctors and nurses are on duty, especially at peak periods. This would be accompanied by efforts to enable clinicians to work a standard day, with a few hours extra if needed on occasion, but without encouraging clinicians to work nearly double their contracted hours each week. Second, the Hong Kong SAR Government could work to educate the general public about the reasons for attending an ED so as to reduce unnecessary patient presentations. In addition, education programmes for the general public, and within the waiting areas of EDs, could strive to realign patient expectations, teaching patients how healthcare services are delivered in hospitals so that patient expectations are in keeping with actual practice.

To summarise, this paper argues that hospitals should develop and implement best-practice policies and educational programmes for clinicians that focus on the following:

1. Understanding the primary causes of communication problems in EDs
2. Accepting the tenets and practices of patient-centred care
3. Establishing clear and consistent knowledge transfer procedures (especially for triage and handover)
4. Lowering the patient-to-clinician ratio in order to create the conditions in which clinicians can engage in effective communication with patients and other clinicians.

Despite the inherent challenges in communication between clinicians and patients in EDs, these issues are critical to resolve because a patient’s satisfaction following an ED visit is a key indicator of that patient’s recovery and subsequent well-being [11, 29]. The key points of transfer for medical knowledge (e.g. triage and handover) clearly deserve further research attention in a range of healthcare contexts.

This research project’s analysis of clinicians’ perspectives highlights the complexity of communication in EDs, the challenges faced by clinicians and the key areas for improvement. The data and subsequent analysis in this research, as well as the suggestions for future clinician education programmes and hospital policies, forge a path towards clearer, safer and more rewarding clinician-patient communication in EDs. These efforts are highly likely to reduce adverse events, raise the level of patient satisfaction, improve patient outcomes and enhance clinician job satisfaction. In this sense, patient-centred policies and clinician communication programmes are time-efficient, cost-effective forms of intervention to improve the quality of care in EDs.
